# Personalizing core decompression grafting technique for osteonecrosis of the femoral head: calculating the volume of bone resected and adjunct volume required to fill the defect

**DOI:** 10.1186/s13018-025-05606-5

**Published:** 2025-03-03

**Authors:** Reza Bergemann, Alexandra Massey, Steven Tommasini, Daniel Wiznia

**Affiliations:** 1 Orthopaedics and Rehabilitation, Yale School of Medicine, Yale University, New Haven, CT USA; 2 Biomedical Engineering, Yale School of Engineering and Applied Sciences, Yale University, New Haven, CT USA; 3 Mechanical Engineering and Material Sciences, Yale School of Engineering and Applied Science, Yale University, New Haven, CT USA

**Keywords:** Osteonecrosis, Bone graft, Core decompression

## Abstract

**Background:**

Osteonecrosis of the femoral head can be a debilitating disease leading to collapse of the femoral head and the subsequent need for a hip arthroplasty. Core decompression has emerged as a leading treatment to prevent collapse. Adjunctive therapies, such as bone graft, bone marrow aspirate concentrates, or synthetic bone substitutes are utilized to promote native bone regeneration. Determining the amount of bone resected and the volume of adjunct required is challenging, especially with newer minimally invasive reamers. Under- or over-filling the defect may impact progression of the disease or cause morbidity.

**Surgical technique:**

We introduce a mathematical method to be utilized intraoperatively to calculate the volume of bone resected during core decompression with an expandable reamer. This method approximates the core decompression defect as two cylinders using measurements that can be easily taken during the procedure and can be adapted for use with any of the expandable reamer systems available. Using this technique, surgeons can calculate the size of the defect created, which can be used to personalize the amount of adjunct delivered to each patient.

**Conclusions:**

When adjunctive therapies are used with core decompression to treat ONFH, care must be taken when filling the core decompression defect to avoid under- or over-filling the defect, potentially increasing the risk of complications or reducing the efficacy of the procedure. We provide a simple worksheet that can be used by surgeons to help determine how much adjunct should be used.

## Introduction

Osteonecrosis of the femoral head (ONFH) is a degenerative disease characterized by regions of dead bone within the femoral head, due to impaired microvascular supply [1–3]. A number of risk factors have been identified, including, but not limited to trauma, resulting in mechanical vascular disruption, blood dyscrasias, alcohol and corticosteroid use [4]. Approximately 10,000–30,000 new cases of ONFH are reported annually in the United States. The average age of affected patients is between 20 and 40 years old [5]. Left untreated, ONFH may progress to collapse of the femoral head with destruction of the joint, requiring total hip arthroplasty (THA); such cases may represent up to ten percent of all total joint replacements performed in the United States [6]. Compared to patients with osteoarthritis, patients with ONFH who undergo total hip replacement have worse outcomes such as significantly higher rates of revision surgery as well as perioperative fracture and infection [7].

The leading treatment for early stage ONFH is core decompression, a procedure in which the surgeon drills into the lesion within the femoral head, removing the necrotic bone, reducing intra-osseous pressure and providing pathways for revascularization [8–10]. Simple core decompression has not been found to be superior to other joint preserving therapies; however, over the years, multiple adjunctive therapies have been introduced to accelerate the healing process [11]. Among the earliest of such adjuncts used was autologous bone graft, taken from the proximal femur or iliac bone [12–14]. Synthetic bone substitutes containing calcium sulfate and or calcium phosphate have also been used to provide mechanical support to the bone defect as well as provide a structure that may promote revascularization and new bone deposition [15, 16]. Bone marrow aspirate concentrate (BMAC) is more commonly used, which is theorized to enhance bony healing by increasing the number of osteogenic progenitor cells at the site of osteonecrosis [17, 18]; other variations use allograft bone matrix in addition to BMAC to provide a scaffold to encourage new bone formation [19, 20]. The use of these cell therapies with core decompression have demonstrated reduced pain, and lower rates of progression compared to core decompression alone, in both adults and skeletally immature patients [21–23]. Factors associated with treatment failure include longer symptom duration, higher pain scores and greater hip dysfunction [24].

Historically, core decompression has been performed using Kirshner wires and small diameter drill bits, as well as cannulated drill bits passed over the Kirshner wire. However, with the goal of lowering the risk of iatrogenic fractures, recent advances have led to the use of expandable reamers that can remove large volumes of bone within the focus of the lesion without requiring a large diameter drill tract extending through the lateral cortical entry point. With these expandable reamers, the total volume of bone removed can vary dramatically depending on the type of reamer, the diameter to which the reamer is expanded, and the depth and length to which the expanded reamer is deployed. Thus, appropriately determining the volume of adjunct needed can be challenging. Too little may result in voids, causing structural weakness and increased risk of fracture, as well as poor osteoconductive matrix. Excess can add unnecessary cost to the procedure, and lead to over-pressurization, resulting in entry into the adjacent soft tissue or joint space, where it may cause inflammation and pain or into the vascular system with concern for embolization [25–28]. We believe it is important to know the size of the defect in order to personalize the amount of adjunct delivered for each patient. We introduce a simple mathematical method for approximating the volume of bone removed during the core decompression, allowing surgeons to better personalize the amount of adjunct used for each patient.

## Surgical technique

Here we describe how to calculate the volume of the core decompression defect created using an expandable reamer system. There are several expandable reamer systems on the market, and this calculation has been developed so that it is easily adapted, regardless of the reamer system. Most expandable reamers have a shaft diameter (SD) and a reamer diameter (RD). Some expandable reamers have a fixed diameter when deployed, while others have a variable cutting diameter (Table 1). As such, the exact steps and surgical instruments used may vary to accommodate the different designs. In general, patients at our institution are selected for core decompression if they are symptomatic and found to have ARCO stage I or II avascular necrosis on imaging, without signs of femoral head collapse or osteoarthritis. All core decompression cases receive adjunctive bone marrow aspirate concentrate with either demineralized bone allograft or synthetic bone substitute.

**Table 1:** Common expandable reamers

Tool	Shaft diameter (mm)	Maximum expanded diameter (mm)
Arthrex IOBP decompression device	3.3	7.0
Arthrex flipcutter III	3.5	12.0
Arthrex AVN reamer	5.0	18.0
Wright medical X-ream	9.0	21.0

Surgeons typically utilize fluoroscopy to aim a Kirshner guide wire, which is advanced from the lateral aspect of the proximal femur towards the area of the necrotic lesion. We recommend utilizing a 3D navigated approach for more accurate targeting [20, 29]. Once the tip of the Kirshner wire has reached the avascular region and its location has been confirmed by fluoroscopy, instrumentation is utilized to prepare the drill tract for the expandable reamer. Some systems utilize a cannulated drill bit that is advanced over the Kirshner to provide a wider drill tract for the expandable reamer shaft; other reamers can be advanced within a Jamshidi needle cannula. The expandable reamer is advanced to the superior aspect of the necrotic region under fluoroscopy. At this point, the total length (TL) of the expandable reamer shaft is then recorded. The reamer is expanded to the desired size and reaming is performed through the extent of the ONFH lesion. The total distance reamed at maximum reamer expansion (RL) is recorded. The expandable reamer is then retracted and removed.

**Fig. 1:**
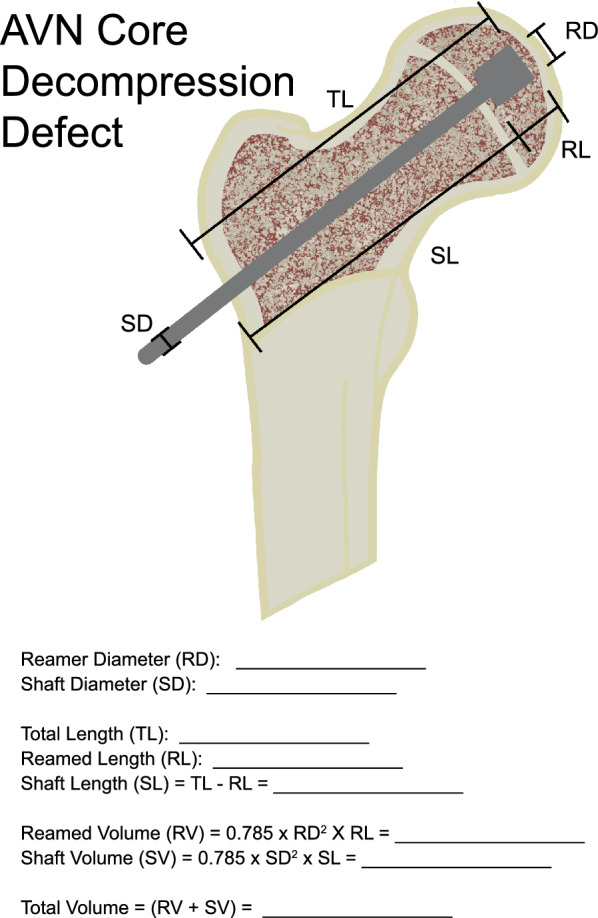
Worksheet used to calculate core decompression defect size

The total volume of bone resected is then calculated using a worksheet as an aid (Fig. 1). The volume of bone resected is approximated by two cylinders: one representing the amount of bone removed by the reamer shaft, and the second representing the bone removed by the expanded reamer. Thus, the reamed volume (RV) = π/4 × RD^2^ x RL, and the shaft volume (SV) = π/4 × SD^2^ x SL, where RD and SD are the diameter of the expandable reamer and the shaft diameter respectively, RL is the length removed by the expanded reamer and SL is the total length minus the reamed length RL. The total volume is thus the sum of the reamed volume (RV) and the shaft volume (SV). This method is agnostic of the location of the lesion, as it only depends on the dimensions of the expandable reamer used during the procedure, and the length for which it is deployed and the depth at which it is deployed. While it is uncommon to perform multiple passes with expandable reamers, if multiple cores are taken in different areas of the femoral head, the calculation can be performed independently for each, and the total volume summed. However, if different cores overlap substantially, the volume calculated will overestimate the size of the defect and should not be used.

In our practice, we mix bone marrow aspirate concentrate (BMAC) with demineralized bone allograft or synthetic bone substitute; accordingly, bone marrow aspirate is collected from the iliac crest and concentrated (Thus producing BMAC). The BMAC is then mixed with the bone allograft at an approximate ratio of one-part BMAC to two-part allograft to achieve a consistency that allows for the allograft to be injected through a Jamshidi cannula. The exact method for preparation will vary depending on the adjunctive therapy chosen. We have found that the volume noted by the manufacturer can vary widely when compared to the volume after the bone graft has been mixed with BMAC. Therefore, prior to injecting the adjunct through the cannula, its volume should be measured with a 5 cc or 10 cc syringe to ensure that the total volume required has been achieved. We have found the exact volume of adjunct needed to fill the defect varies depending on the type of adjunct used (i.e. synthetic bone substitute or demineralized bone chips). In general, we have found an additional 25–50% volume to adequately pack the defect without substantial extravasation. The prepared adjunct is then injected via the Jamshidi needle cannula under fluoroscopic monitoring to ensure appropriate filling of the defect. If the defect appears uniformly dense compared to surrounding bone on fluoroscopy, we consider the defect to be filled. If there are patchy areas or areas less dense than surrounding bone, further injection is needed to fully pack the defect.

### Case 1

**Fig. 2:**
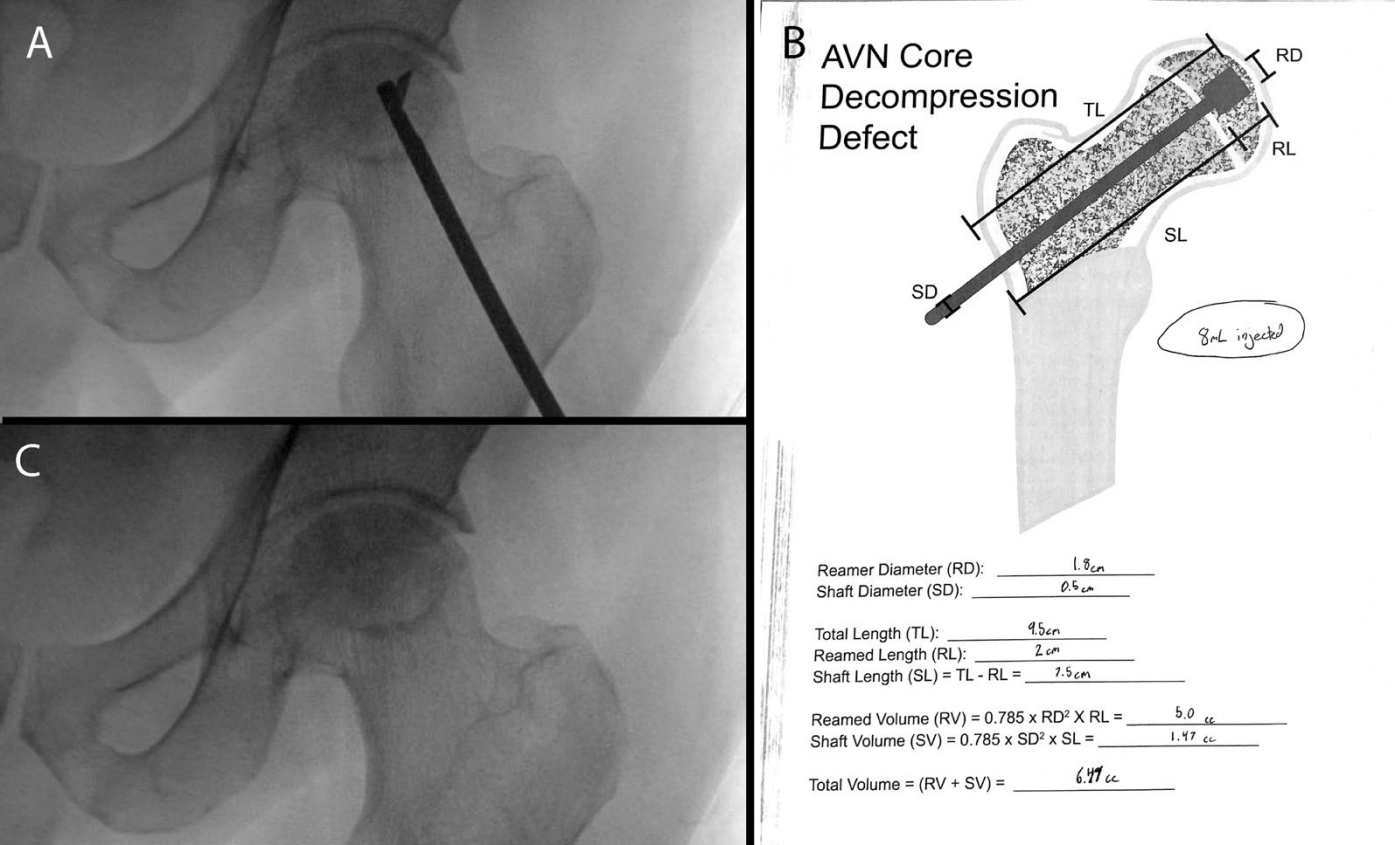
Case 1. **A** Deployed expandable reamer. **B** Worksheet used to calculate required bone graft volume; volume of bone graft injected was approximately 1.25 × that calculated. **C** Implanted bone graft, with complete filling of core decompression defect

The patient is a 46 year old man who presented with complaint of bilateral hip pain, greater on the left side, which reportedly began shortly after being hospitalized for a COVID-19 infection. On imaging, he was found to have ARCO stage II avascular necrosis of the left femoral head, and stage III avascular necrosis of the right femoral head. The patient underwent 3D navigated core decompression of the left femoral head using the Medtronic Stealth System [20]. Bone marrow aspirate was collected from the left anterior iliac crest and was concentrated with an automated centrifuge concentrating system. An expandable reamer (Arthrex AVN Reamer) was used to decompress the AVN lesion (Fig. 2A). Bone marrow aspirate concentrate was mixed with allograft demineralized bone matrix (Arthrex AlloSync demineralized bone matrix). Using the worksheet, the volume of the defect was calculated to be 6.47 mL (Fig. 2B). Approximately 8 mL of mixed adjunct was injected through a Jamshidi needle, approximately 1.25 times the calculated defect. After injection, intraoperative fluoroscopy revealed complete filling of the core decompression defect, without significant extravasation (Fig. 2C). The patient was discharged the following day and was made weightbearing as tolerated. At follow-up five months later, the patient reported almost complete resolution of pain and significantly improved mobility of the left hip. Radiographically, there was no evidence of fracture or collapse of the left hip.

### Case 2

**Fig. 3:**
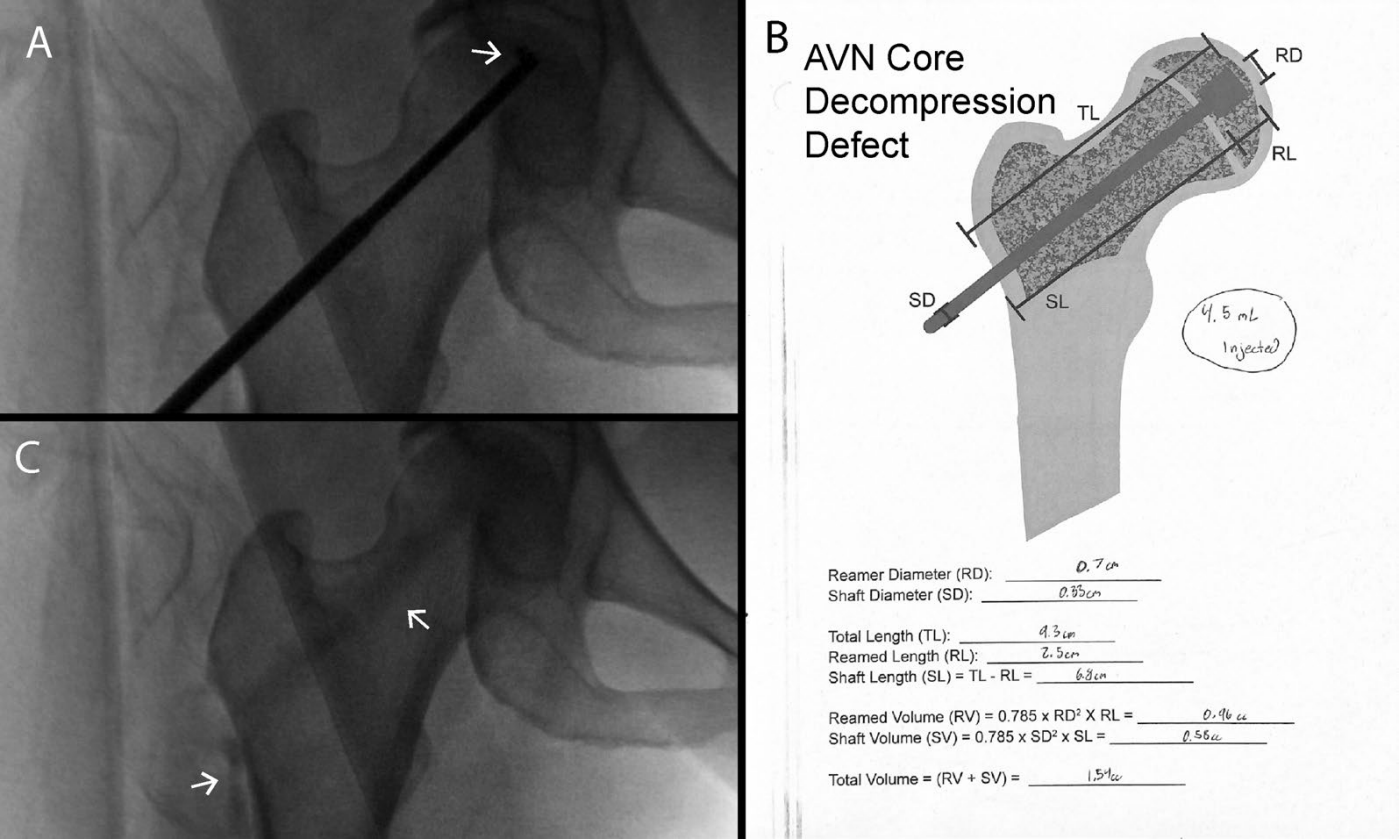
Case 2 with over-injection. **A** Deployed expandable reamer. **B** Worksheet used to calculate required bone graft volume; note volume of bone graft injected was approximately 2 × that calculated. **C** Implanted bone graft, with extravasation into femoral neck and adjacent soft tissue

The patient is a 50 year old women who presented with complaint of right hip pain. She was found on imaging to have ARCO stage II avascular necrosis of the right femur. Risk factors for avascular necrosis included extensive corticosteroid exposure, secondary to Still’s disease. The patient underwent 3D navigated core decompression using the Medtronic Stealth System. Bone marrow aspirate was collected from the right anterior iliac crest and was concentrated with an automated centrifuge concentrating system in the OR. An expandable reamer (Arthrex IOBP decompression device) was used to decompress the AVN lesion (Fig. 3A). Using the worksheet, the volume of the defect was calculated to be 1.54 mL (Fig. 3B). Approximately 5 mL of bone marrow aspirate concentrate was mixed with a 5 mL kit of synthetic bone substitute (Arthrex BoneSync calcium phosphate cement). The mixed adjunct was injected through Jamshidi needle until resistance was felt. The total volume injected was 4.5 mL, approximately three times the calculated defect volume. After injection, intraoperative fluoroscopy revealed significant extravasation of adjunct out of the lateral cortical defect and into cancellous bone of the metaphysis (Fig. 3C). Post-operatively, the patient was weightbearing as tolerated. At six weeks follow-up, the patient reported reduced pain with occasional soreness after prolonged standing. She was found on X-ray to have good incorporation of the graft without fracture or subchondral collapse.

## Discussion

Core decompression has emerged as the leading treatment for ONFH to prevent disease progression leading to femoral head collapse and the need for total hip replacement. The use of adjunctive therapy with core decompression may promote revascularization and enhance bony healing, and provide improved structural stability [30]. However, it is typically unclear what the appropriate volume is required to fill the void left by the core decompression procedure, and adjuncts can add significant cost to the procedure. Traditionally, filling of the osteonecrotic defect has been performed until there is resistance to continuous injection. This is not always a failsafe method, as observed in case 2. In this case, bone graft was injected until resistance was felt. After injection, intraoperative fluoroscopy revealed significant extravasation of adjunct out of the lateral cortical defect and into cancellous bone of the metaphysis (Fig. 3C). The worksheet was used to calculate the volume of the defect, and it was determined approximately three times the defect volume was injected. Had the size of the core decompression defect been known, the surgeon may have opted to inject less substitute even though the typical resistance was not detected.

While the extravasation was benign in the case above, over-injection may increase morbidity of core decompression. Synthetic bone substitutes have been found to cause pain and inflammation in soft tissue [25]. Extravasation may be particularly concerning in patients with later stage osteonecrosis with subchondral fractures, as these fractures may provide a pathway for the adjunct to enter the joint space, where they could accelerate destruction of the joint space and cause significant pain. Additionally, fat embolization is considered a risk of core decompression [26, 28]. Overfilling may increase this risk as increased intramedullary pressure has been found to be a major factor in the entry of fat into the vascular system [31, 32]. Beyond complications, excessive use of adjuncts can also significantly increase the cost of the procedure: at our institution, 5 cc of demineralized bone matrix costs approximately $1000 while synthetic bone substitute costs approximately $1500 for an equivalent amount. It is thus crucial to avoid over-injection of adjunct. While there is less prior literature on under-filling, we believe it should also be avoided for the following reasons: voids left within the defect may limit the efficacy of core decompression, by leaving areas that do not receive the osteogenic properties of the adjuncts used during core decompression. Additionally, voids may also increase the risk of perioperative fracture, as they will create localized stress risers. Given the need to appropriately pack the defect with adjunct, we feel it is important for surgeons to personalize the volume of bone graft for each patient. The worksheet provides surgeons with an estimate of the size of the defect, enabling better personalization.

The mathematical method for determining the appropriate volume of adjunct for core decompression is limited by the fact our calculation is an estimation of the shape created by the expandable reamer. While we assume the shape to be essentially two cylinders of different volume, this may not precisely represent the defect, and thus the calculated volume may differ slightly. Nonetheless, we feel this method is sufficient to provide an individualized estimate of the core decompression defect and thus provide surgeons with a basis to personalize their core decompression procedure. While we have not experienced any complications since starting to use this technique, more research is needed on the long-term outcomes for patients.

## Summary

Determining the appropriate amount of graft material can be challenging, and if not calculated accurately, may lead to impaired healing of the defect, wasted graft material or increased risk of complications such as fat embolism, inflammation of adjacent soft tissue, or extravasation into the joint space. We introduce a simple mathematical method for calculating the core decompression defect. By approximating the volume of the defect created during core decompression, a surgeon can estimate the size of the defect, which can then be incorporated into the operative plan to personalize the procedure.

## Data Availability

No datasets were generated or analysed during the current study.

## Abbreviations

ONFH: Osteonecrosis of the femoral head
THA: Total hip arthroplasty
BMAC: Bone marrow aspirate concentrate
